# The Regulatory Network of Sturgeon Chondroitin Sulfate on Colorectal Cancer Inhibition by Transcriptomic and Proteomic Analysis

**DOI:** 10.3390/ijms22179395

**Published:** 2021-08-30

**Authors:** Ruiyun Wu, Qian Shen, Guangyue Li, Pinglan Li, Nan Shang

**Affiliations:** 1 Key Laboratory of Functional Dairy, Ministry of Education, College of Food Science and Nutritional Engineering, China Agricultural University, Beijing 100083, China; wry0814@cau.edu.cn; 2 Department of Biology, Rhodes College, 2000 North Pkwy, Memphis, TN 38112, USA; shenq@rhodes.edu; 3 Key Laboratory of Saline-Alkali Vegetation Ecology Restoration, Ministry of Education, College of Life Science, Northeast Forestry University, Harbin 150006, China; guangyuel@nefu.edu.cn; 4 College of Engineering, China Agricultural University, Beijing 100083, China; 5 Key Laboratory of Precision Nutrition and Food Quality, Department of Nutrition and Health, China Agricultural University, Beijing 100083, China

**Keywords:** sturgeon chondroitin sulfate, colorectal cancer, RNA-seq, cell cycle, apoptosis, tumor xenograft

## Abstract

Chondroitin sulfate (CS) is a food-derived bioactive substance with multiple biological functions, which exists in animal cartilage and/or bone. Sturgeon, a type of cartilaginous fish, is rich in CS. Our recent study demonstrated the effect of sturgeon chondroitin sulfate (SCS) on reducing colorectal cancer cell proliferation and tumor formation. However, the molecular mechanisms of its anticancer activity remain unknown. In this study, the cell proliferation assay and flow cytometric analysis were used to examine the cell viability and apoptosis of colon cancer cell HT-29 cells and normal colonic epithelial cell NCM460 cells. Transcriptomic and proteomic studies were used to identify the main targets of SCS. SCS showed little effect on the genes/proteins expression profile of NCM460 cells but more sensitive to HT-29, in which 188 genes and 10 proteins were differentially expressed after SCS treatment. Enrichment analysis of those genes/proteins showed that the majority of them are involved in DNA replication, cell cycle progression and apoptosis. Quantitative RT-PCR and Western blot were used to determine essential genes/proteins and networks targeted by SCS to exert inhibiting the development of colorectal cancer function. This study provided great insights into developing food-derived novel therapeutics for colorectal cancer treatment.

## 1. Introduction

Colorectal cancer (CRC) is a common digestive tract cancer with increasing incidence rate and mortality rate worldwide in recent years [1]. However, due to the long incubation period of CRC, which is generally 7–10 years, it is usually found during the middle and advanced stage [2,3]. Currently, chemotherapy, surgical resection and antibiotics are commonly used as standard clinical treatments. These methods have limited efficacy in preventing tumor progression and always lead to considerable drug resistance and poor prognosis [4,5]. Therefore, it is urgent to develop new therapeutic approaches to treat this fatal cancer.

Many natural compounds exhibited anti-cancer activity [6]. Many studies have shown that natural bioactive compounds can be used as drugs or adjuvants for tumor treatment with fewer side effects [7]. As a natural polymer, chondroitin sulfate (CS) is the main active component in animal cartilage. It is mainly composed of glucuronic acid and galactosamine repeatedly linked by alternately β-glycosidic bond. CS has a variety of biological activities such as regulating cell viability and maintaining the integrity of tissue structure [8,9,10,11]. They have been widely used for medical and health purposes, such as the treatment of osteoarthritis, cardiovascular diseases and tissue regeneration. In recent years, CS has drawn increasing attention due to its antitumor activity. For example, oyster CS can inhibit the development of cancer by activating MAPK signaling pathway [12]. In addition, the in vivo and in vitro data from our previous study show that CS can inhibit the proliferation of HCT-116 cells by inducing apoptosis, which suggests that CS has antitumor activity against colorectal cancer [13].

Although CS has been widely used, like many other natural compounds, the anticancer mechanism of CS remains elusive. In this study, we used HT-29 cells as the CRC cancer model cells to investigate the antitumor mechanisms of a hybrid sturgeon (*Acipenser schrenckii* × *Huso dauricus*)-derived chondroitin sulfates (SCS). RNA- seq and 2-DE were used to identify the differentially expressed genes/proteins after SCS treatment and the regulatory network of SCS inhibiting the development of colorectal cancer was predicted. Meanwhile, HT-29 cells were also used to induce xenograft tumors in mice and to validate the in vivo anti-cancer effect of SCS. The key genes/proteins in networks targeted by SCS to inhibit the occurrence of CRC were detected by quantitative RT-PCR and Western blot as well as to study the underlying molecular mechanisms.

## 2. Results and Discussion

### 2.1. SCS Inhibits the Proliferation of Colorectal Cancer Cells

**Figure 1:**
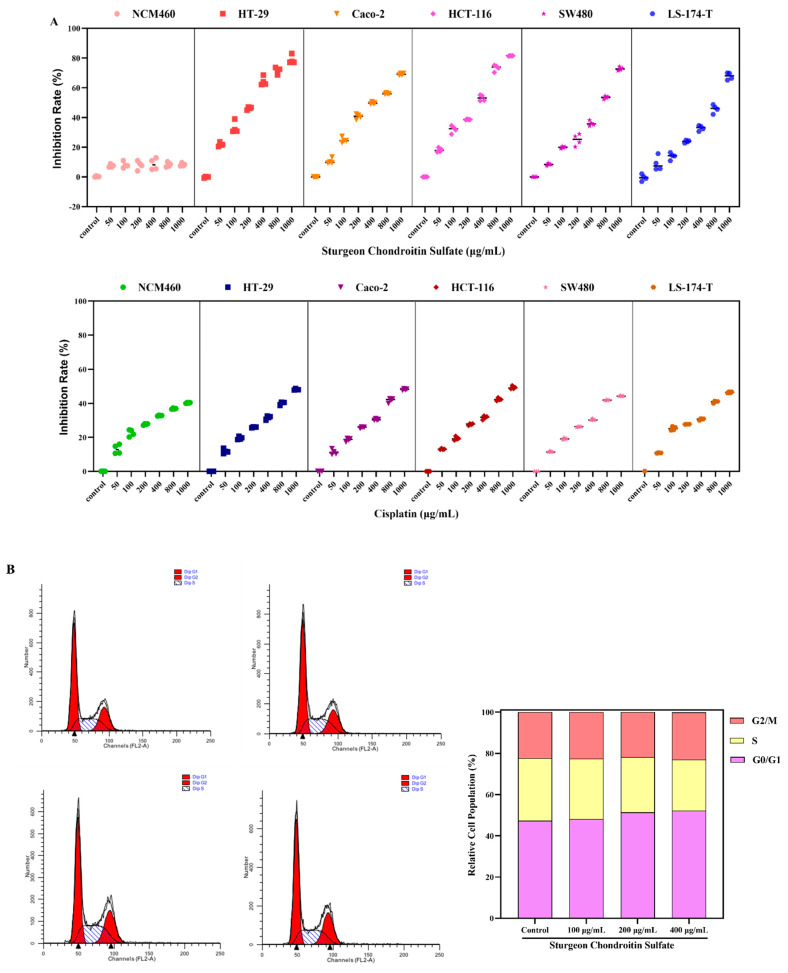

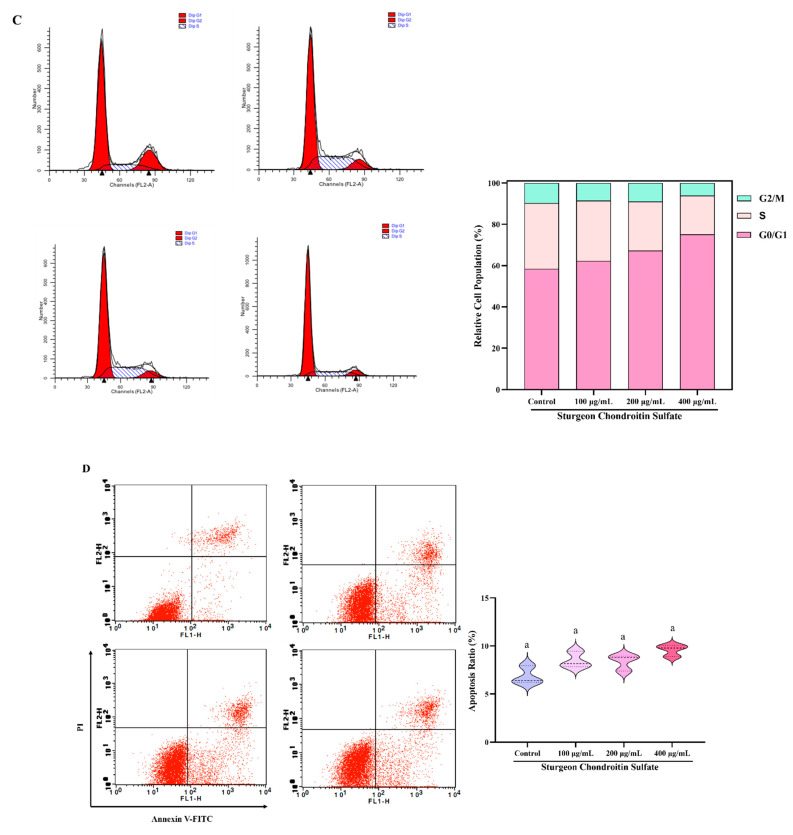

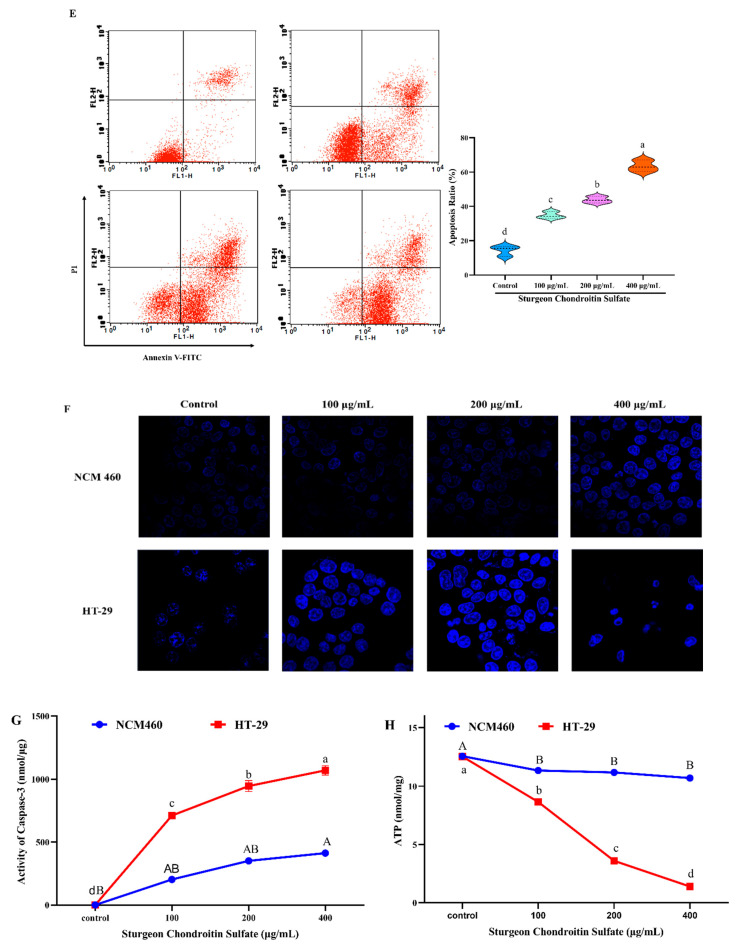
Effect of chondroitin sulfate (SCS) on cell proliferation in colon cancer cells and normal intestinal epithelial NCM460 cells. (**A**) Colon cancer HT-29, Caco-2, HCT-116, SE480, LS-174-T cells and normal intestinal epithelial NCM460 cells were treated with different concentration (50, 100, 200, 400, 800, and 1000 μg/mL) of SCS and positive control cisplatin for 24 h. Cell viability was measured by CCK8 cell counting assay and the results were expressed as inhibition rate (%) compared to untreated cells; (**B**) Effect of SCS on cell cycle arrest in normal intestinal epithelial NCM460 cells, (**C**) Effect of SCS on cell cycle arrest in colon cancer HT-29 cells. The percentages of cells in each phase (G0/G1, S, G2/M) were calculated by cell cycle assay kit and flow cytometer; (**D**) Effect of SCS on cell apoptosis in normal intestinal epithelial NCM460 cells; (**E**) Effect of SCS on cell apoptosis in colon cancer HT-29 cells. The apoptosis ratios (%) were calculated by cell apoptosis assay kit and flow cytometer; (**F**) Representation images of apoptotic bodies, the apoptotic bodies were identified by immunofluorescence microscopy; (**G**) After SCS treatment, the expression of ATP in NCM460 cells and HT-29 cells were affected; (**H**) After SCS treatment, the expression of Caspase-3 in NCM460 cells and HT-29 cells were affected. All results are expressed as mean ± SD (*n* = 3). Different superscript letters for each column indicate significant differences (*p* < 0.05) as compared to every other group.

To investigate the anti-proliferative effect of SCS, the cell counting assay (CCK-8 kit) was used to quantify the viability of multiple cell lines (icludingHT-29, Caco-2, HCT-116, SW480, SL-174-T, and NCM460) after SCS treatment. The SCS can inhibit all colorectal cancer cell lines growth (Caco-2, HCT-116, SW480, SL-174-T) in a dose dependent manner (Figure 1A). In contrast, SCS showed little cytotoxicity on normal colon cell line NCM460 (8.77 ± 0.02%). These results indicate that the inhibitory activity of SCS is specific to colon cancer cells, and has no toxicity to normal colon cells NCM460. At the same time, the IC50 values of SCS for each cell were 7751 μg/mL (NCM460), 331.3 μg/mL (HT-29), 341.7 μg/mL (Caco-2), 352.4 μg/mL (HCT-116), 368.7 μg/mL (SW480) and 397.7 μg/mL (LS-174-T). IC50 of positive control Cisplatin were 1026 μg/mL (NCM460), 917.5 μg/mL (HT-29), 914.7 μg/mL (Caco-2), 862.2 μg/mL (HCT-116), 958.0 μg/mL (SW480), and 903.1 μg/mL (LS-174-T), respectively. The IC50 results show that the IC50 values of SCS on five kinds of colorectal cancer cells were close, indicating that SCS had a general inhibitory effect on the proliferation of colorectal cancer, and had little toxicity to normal intestinal cell NCM460.

### 2.2. SCS Induces Cell Cycle Arrest of HT-29 Cells

To investigate whether the anti-proliferative effect of SCS is due to cell cycle arrest, the proportion of cells from different cell cycle phases (including G0/G1, S and G2) was determined using flow cytometry. SCS treatment resulted in an increase in G0/G1 phase cell proportion in HT-29 cell (Figure 1C). In contrast, SCS treatment did not alter the proportion of cells from different phases of the cell cycle in normal colon cell line NCM460 (Figure 1B).

The period from mitosis to DNA replication, also known as G1 phase of synthesis [14,15], during which RNA and ribosomes are mainly synthesized [16,17]. This phase is characterized by active metabolism, rapid synthesis of RNA and protein and significant increase in cell volume. This phase is to prepare material and energy for the S phase where DNA replication occurs [18,19]. Therefore, prolonging G0/G1 phase can effectively delay cells from entering S phase, block DNA synthesis and decrease proliferation activity. A number of anti-cancer drugs works by inducing cell cycle arrest such as Neuronal pentraxin [20] and ZNF674-AS1 [21]. Our results suggest that causing cell cycle arrest is part of the SCS antitumor mechanism.

### 2.3. SCS Induces Apoptosis

Flow cytometry analysis was performed to quantify the percentage of apoptotic cells after SCS treatment (Figure 1D,E, Table S1). In HT-29, the ratio of apoptotic cells was up to ~50% after 400 μg/mL SCS treatment, whereas SCS treatment had little effect on NCM460 cells. In addition, immunofluorescence microscopy further confirmed the stimulatory effect of SCS on the apoptosis of HT-29 (Figure 1F). Compared with NCM460 cells, the HT-29 cells exhibited a marked increase in apoptosis, exhibiting pronounced nuclear condensation, DNA fragmentation and the formation of apoptotic bodies.

The lack of apoptosis is an important feature of tumor cells [22,23]. Singh (2019) has shown that the apoptosis level of colorectal cancer and adenoma is significantly reduced [24]. The current treatment methods mainly focus on promoting the apoptosis of cancer cells [25]. Our results indicate that SCS can induce apoptosis of colon cancer HT-29 cells with little impact on normal colon cells.

### 2.4. SCS Activates the Expression of Apoptotic Enzymes

Since our results show that SCS can induce apoptosis in colon cancer cell line, we sought to determine whether SCS activate specific enzymes involved in cell apoptosis. Caspase is a family of proteases that play an important role in the process of apoptosis. Caspase 3 belongs to CED-3 subfamily of caspase family. It is a key enzyme in the process of apoptosis, which is called apoptosis executive enzyme [26,27]. The high expression of Caspase-3 indicates the occurrence of apoptosis [28]. When compared with control, the Caspase-3 in SCS-treated HT-29 cells was increased, and a reduction of more than 1051.23 ± 0.07 nmol/μg on 400 μg/mL, NCM460 was only 412.07 ± 0.11 nmol/μg (Figure 1G). These results indicate that SCS can induce apoptosis of colorectal cancer HT-29 cells, which is consistent with the results of annexin V-FITC/PI double staining method (Figure 1E).

ATP, as the most important energy molecule, plays an important role in various physiological and pathological processes of cells [25]. The change of ATP level affected the function of cells. Generally, the level of ATP decreases when the cells are in apoptosis, necrosis or some toxic state [29]. After SCS treatment, the cell ATP activity is shown in Figure 1H. SCS did not cause the change of ATP in NCM460 cells, but it could significantly decrease the ATP value in HT-29 cells. Under 400 μg/mL treatment, the ATP value in HT-29 cells decreased from 12.57 ± 0.04 nmol/mg to 1.42 ± 0.09 nmol/mg. Our results show that SCS can deplete ATP levels and increase the production of Caspase-3 in HT-29 cells, indicating that SCS treatment causes apoptosis.

### 2.5. Transcriptomic and Proteomic Analysis of SCS Treated Cells

#### 2.5.1. Identification of Differentially Expressed Genes and Protein in Cells after SCS Treatment

RNA-seq was used to analyze differentially expressed genes in HT-29 and NCM460 cells in response to SCS treatment (100 μg/mL treatment for 24 h). We analyzed all differentially expressed genes in HT-29 cells (log_2_-FoldChange > 1.5, *p* < 0.01) and found a total of 187 differentially expressed genes related to cell proliferation, in which 45 genes were up-regulated and 125 genes were down-regulated. The RNA-seq dataset of this study is available in the Gene Expression Omnibus (GEO) with an accession number of GSE179862.

**Figure 2:**
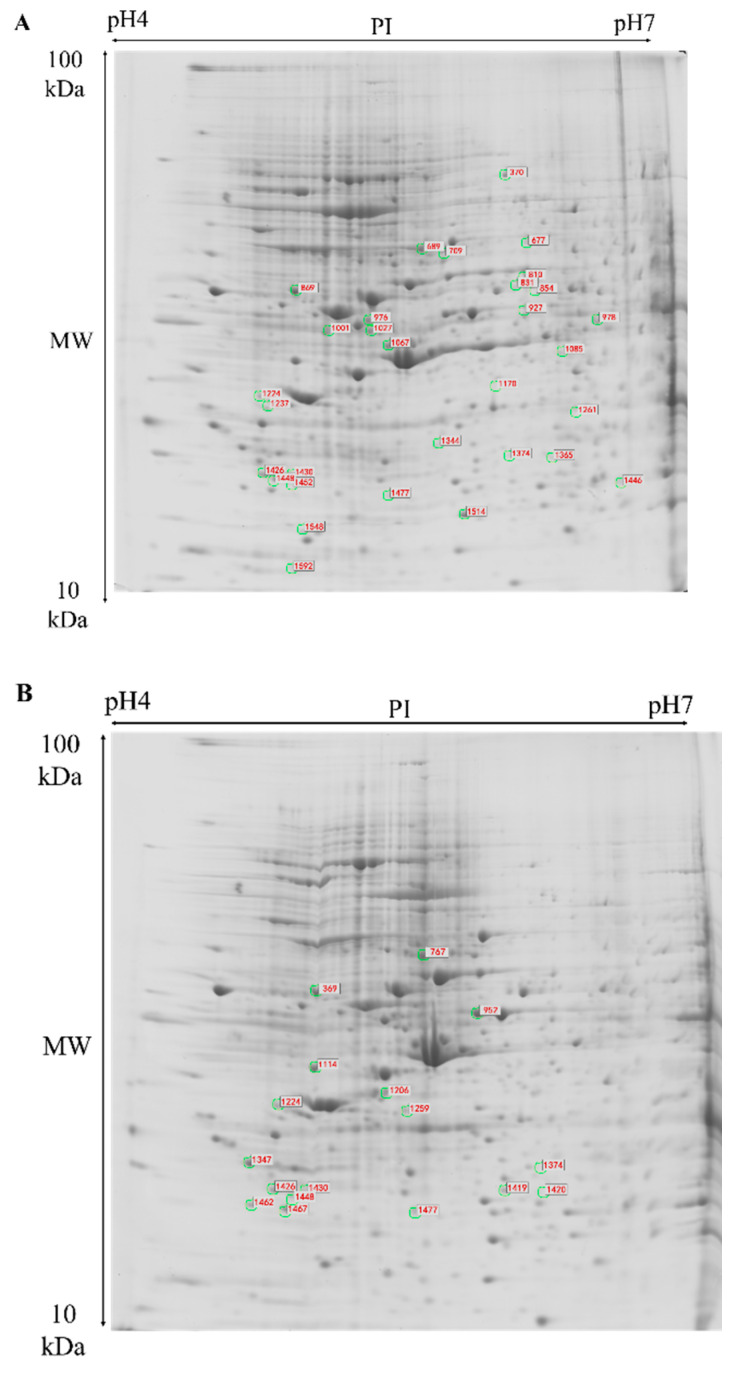
Two-dimensional electrophoresis of the total-cell proteins of HT-29 cells (**A**) and NCM460 cells (**B**) treated with SCS. Differentially expressed protein spots of cells treated with SCS were numbered according to the numbering in Table 2.

Meanwhile, two-dimensional electrophoresis (2-DE) was performed to identify the differentially expressed proteins in HT-29 and NCM460 cells after SCS treatment. Two spots were changed significantly on NCM460 cells among all differentially expressed proteins. In HT-29 cells, through differential analysis with PD Quest software, 10 differential spots were identified by a factor of 1.5-fold (*p* < 0.05) in the SCS treatment group (100 μg/mL) as compared to the control group on the 4-7 NL pH range gels. Most of them are involved in cell cycle, apoptosis, and protein metabolism in cancer cells, which was consistent with the observation that HT-29 cells proliferation activity was inhibited after SCS treatment (Figure 2A,B). These spots were successfully identified by MALDI-TOF MS/MS (Table 1), and the identification rate of MS was 100%.

#### 2.5.2. Impact of SCS Treatment on Metabolic Pathways

**Figure 3:**
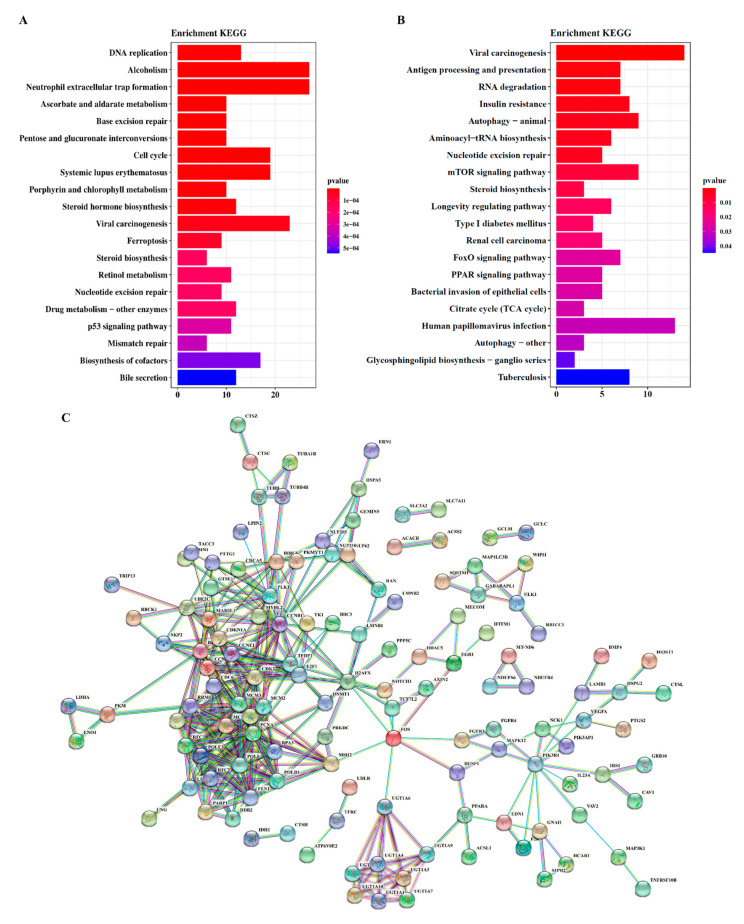

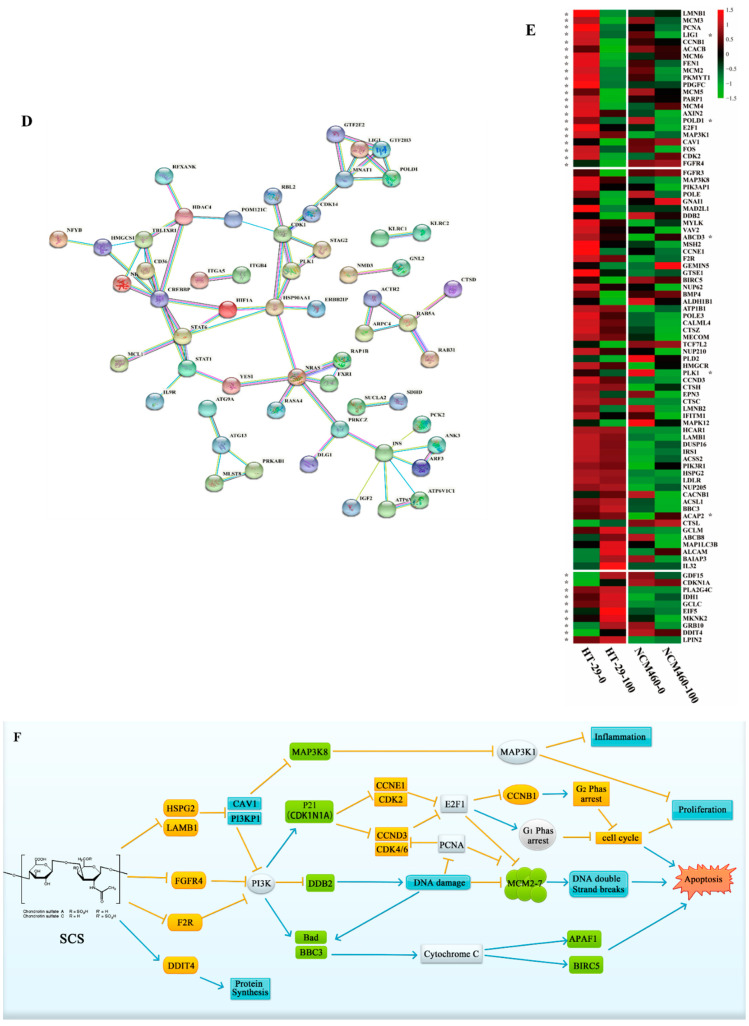
Comparative and integrative analyses of the two RNA-seq datasets from SCS-treated HT-29 cells and NCM460 cells. (**A**) The analyses of the genes with reversely changed expression by the KEGG enrichment analyses in HT-29 cells; (**B**) The analyses of the genes with reversely changed expression by the KEGG enrichment analyses in NCM460 cells; (**C**) The gene-gene interaction network in response in HT-29 cells treated with SCS; (**D**) The gene-gene interaction network in response in NCM460 cells treated with SCS; (**E**) Heat maps show that displayed altered expression of genes in HT-29 cells and NCM460 cells after SCS treatment. Among the 88 genes related to cell cycle and apoptosis pathway, 32 genes in HT-29 cells were significantly different after SCS treatment (fold change > 1.5, *p*-value < 0.05), while only 5 genes in NCM460 were significantly different, * indicates *p*-value < 0.05; (**F**) Regulatory network of SCS inhibiting the development of colorectal cancer was predicted.

To further investigate the key pathways involved in response to SCS treatment, we analyzed all differentially expressed genes after SCS treatment in both HT-29 and NCM460 cells using the KEGG pathway enrichment analysis. We selected the top 10 pathways that showed significant enrichment (Figure 3A,B) and the gene interaction network was shown in Figure 3C,D. Two volcanic maps were generated using differential gene data (Figure S1A,B) and GO function classification (Figure S2A,B). With the gene count in each signaling pathway as a parameter, we observed that the differentially expressed genes were mainly enriched in DNA replication, apoptosis, cell cycle, and PI3K-AKT pathway in HT-29. In particular, the DNA replication, cycle and neutrophil extracellular traps formation pathway exhibited the lower Q-value, indicating its highest enrichment, and similar results were also found in Ellagic acid treatment hepatocellular carcinoma cells [15]. Among them, neutrophil extracellular traps pathway is related to bacteriostasis and anti-inflammatory, and can induce the changes of inflammatory factors, indicating that SCS can regulate the expression of inflammatory related factors in colorectal cancer and has anti-inflammatory effect. Consistently, the GO analyses shown above also revealed that SCS treatment greatly impacted various cellular processes and different cellular parts or organelles. After SCS treatment, differentially expressed genes were mainly enriched in RNA transport, growth hormone synthesis, secretion pathways in NCM460. In addition, the regulatory network of gene interaction was constructed for different genes in HT-29 and NCM460 cells after SCS treatment (Figure 3C,D). The interactions among cell cycle, apoptosis and DNA replication in HT-29 cells were found after SCS treatment whereas no such changes were found in NCM460 cells. The heat map directly displayed altered expression of 88 genes in HT-29 cells after SCS treatment (Figure 3E), which involved in DNA replication, apoptosis, cell cycle, focal adhesion, and antigen processing and presentation. Among the 88 genes related to cell cycle and apoptosis pathway, 32 genes in HT-29 cells were significantly different after SCS treatment (fold change > 1.5, *p*-value < 0.05), including 10 up-regulated genes and 22 down-regulated genes. In NCM460 cells, there were only five genes with significant difference, two genes were up-regulated and three genes were down regulated. There are two differential genes (*LIG1*, *POLD1*) in HT-29 and NCM460. Therefore, SCS has a great impact on apoptosis and cell cycle-related pathways in HT-29 cell line. At the same time, we also analyzed the expression of these genes in NCM460 cells after SCS treatment. We found that the genes involved in apoptosis were not up-regulated in NCM460 cells. The expression of genes involved in DNA replication and cell cycle changed after SCS treatment. For example, the expression of gene *POLD1*, *LIG1* and *PLK1* involved in DNA replication were decreased by 1.81-fold, 1.77-fold, and 1.67-fold, respectively. However, the magnitude of change was significantly lower than that in HT-29 cells (Tables S1 and S2). In addition, although *POLD1* gene is related to DNA replication, the expression change of *POLD1* was associated with autosomal dominant predisposition to benign colonic adenomatous polyps. Similarly, *LIG1* encodes a member of the ATP-dependent DNA ligase protein family, and mainly participates in DNA replication [30]. Therefore, there are still some ambiguities about the function of this gene, which cannot be used as the basis for SCS toxicity to normal colon cell NCM460. The above results show that SCS could block the cell cycle by inhibiting DNA replication, promote apoptosis and inhibit the development of colorectal cancer. Thus, DNA replication, apoptosis and related cell cycle processes are likely the most impacted pathways in HT-29 cells in response to SCS treatment. The metabolic pathways were identified using a *p*-value < 0.05 as the cut-off and 10 differential proteins were analyzed to obtain functional insights into the differences between the proteomes of SCS treated and control group. A total of 10 differentially expressed proteins (Table 1) were involved in transcription and translation, PI3K-AKT signaling pathway, cell cycle, and apoptosis. These results are consistent with our transcriptome data, in which SCS can induce apoptosis and increase *Bad*, *P21* gene expression in HT-29 cancer cells, whereas SCS treatment showed little impact on normal cells NCM460. Collectively, the inhibitory effect of SCS on colorectal cancer could be mainly through the interfering DNA replication and cell cycle and promoting apoptosis. According to the differential expression function prediction of transcriptome and proteome, regulatory network of SCS inhibiting the development of colorectal cancer was predicted (Figure 3F).

### 2.6. SCS Improves Mice Survival and Reduces Damages on Tumor Surrounding Tissues and Organs

After observing the inhibitory effect of SCS on colon cancer cell line, we were interested in determining whether this inhibitory effect occurs in vivo. Meanwhile, we also sought to determine whether genes that were differentially regulated in SCS treated cancer cell line are differentially regulated mouse tumors. At the same time, in order to determine the intragastric concentration, the experiment tested the proliferation of transplanted tumors with different concentrations of SCS (50, 100, 200, 400, 800 and 1000 μg/g). We found that SCS can significantly slow down the proliferation of transplanted tumors at the concentration of 100 μg/g compared with the positive control cisplatin (Figure S4). Therefore, 100 μg/g was selected as a low concentration in the follow-up experiment.

**Figure 4:**
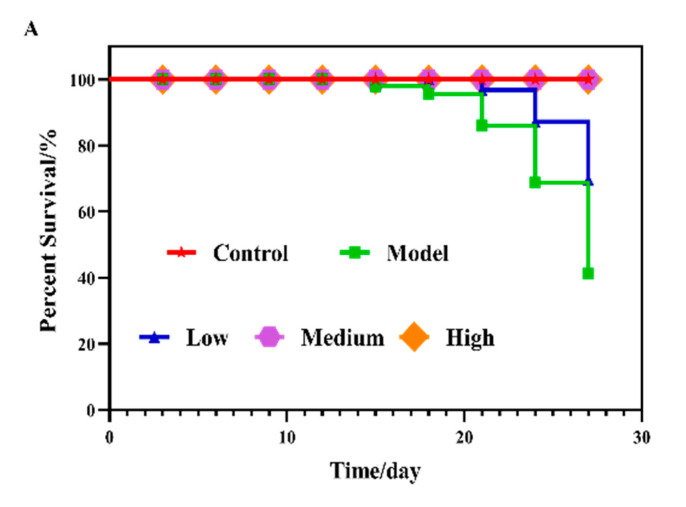

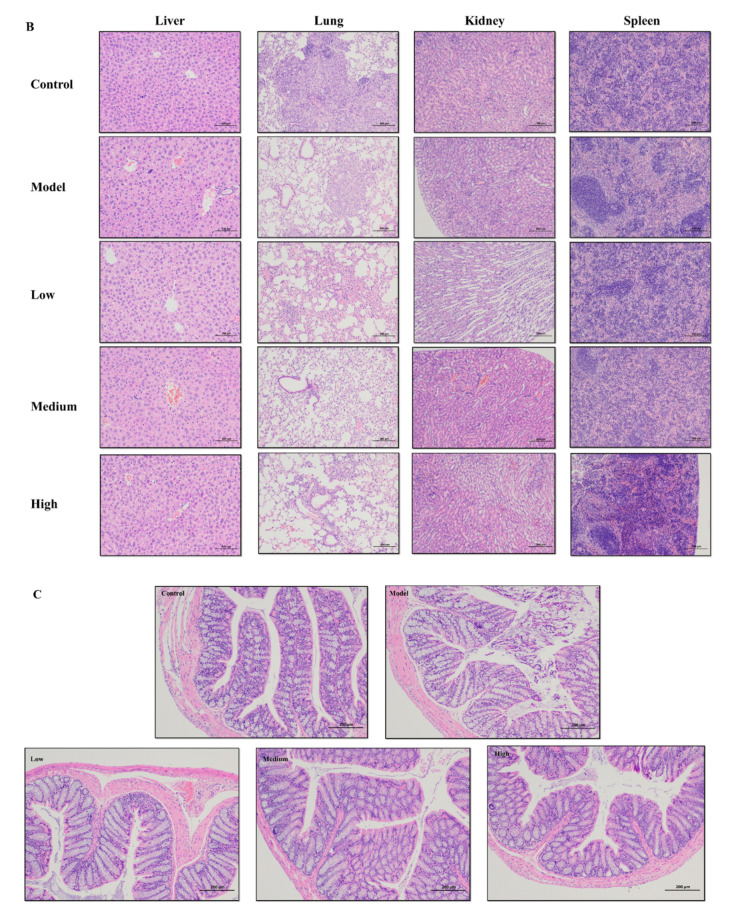

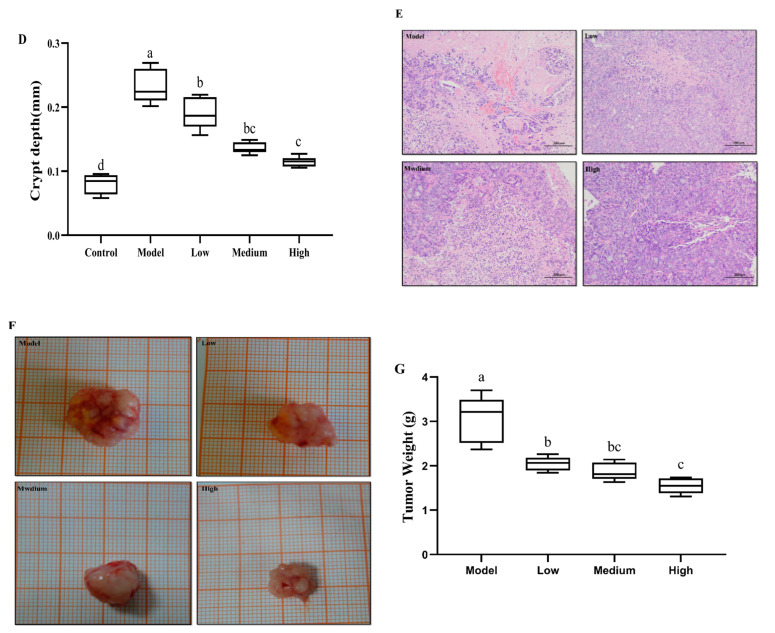
Effect of SCS on tumor in HT-29 xenograft tumor mice model. (**A**) Survival curve; (**B**) Effects on tissues and organs of mice in each group (×100); (**C**) Typical diagrams of HE stained cells in colorectal epithelial tissues with SCS (×100); (**D**) Effects of SCS treatment on the crypt height in colorectal epithelial tissues; (**E**) HE staining of tumor tissue (×100); (**F**) Representation images of tumors from each group; (**G**) Final tumor volume. During the 7-week experiment, the tumor volume of each animal was measured every week and the final tumor volume was measured after sacrifice, all results are expressed as mean ± SD (*n* = 3). Different superscript letters for each column indicate significant differences (*p* < 0.05) as compared to every other group.

To study the inhibitory effect of SCS on tumor growth in vivo, a tumor xenograft model was established in the present study. First, we examined whether SCS confers a survival advantage to mice with colorectal cancer. As shown in Figure 4A. Low dose group (100 μg/g), medium dose group (200 μg/g), and high dose group (400 μg/g) SCS effectively reduced the mortality of HT-29 transplanted tumor mice. The survival rate in the model group was 40%. In contrast, in the high dose group, all mice survived throughout the entire experi-mental period. During administration, the volume change of transplanted tumor of model mice in each group is shown in Figure S3.

The infection of cancer cells can cause damage to other surrounding tissues, thus increasing the mortality rate [31]. Therefore, we also investigated the impact of SCS on multi-organ damages caused by HT-29 cells. As displayed in Figure 4B, SCS treatment reduced histological injury and mitigated the inflammatory cells recruited to liver, lung, and kidney tissues.

### 2.7. Effect of SCS on the Colonic Crypt Depth

After sacrificing the mice, the distal part of colon was removed and the depth of crypt was counted by H&E staining. H&E staining is shown in Figure 4C, which indicates that HT-29 infection increased the depth of colonic crypt, suggesting the tissue damage by cancer cells. The colonic crypt depth of the model group 0.23 ± 0.04 mm was significantly higher than that of the normal group 0.09 ± 0.03 mm. The SCS treatment group could reduce the colonic crypt depth. The high dose group (400 μg/g) was 0.11 ± 0.03 mm, which was restored to the level of the normal group (Figure 4D).

The depth of colonic crypt is the number of cells in the longitudinal half of each crypt, reflecting the balance between the proliferation and apoptosis of intestinal epithelial cells [32]. Studies have shown that a variety of carcinogens or bacteria can induce the change of crypt depth and destroy the balance. A study found that the crypt depth increased significantly in the colorectal cancer model, resulting in mutation accumulation and further development into adenoma and adenocarcinoma. In the dimethylhydrazine (DMH)-induced rat model, a further increase in crypt depth was also observed. In addition, compared with the control group, the depth of crypt decreased by 85% after anti-inflammatory drug sulindac treatment [33,34]. Our results also show that the reduction in the crypt depth is an indication of SCS’s antitumor activity.

### 2.8. SCS Suppresses the Growth of HT-29 Tumor Xenograft In Vivo

After 2 weeks of HT-29 cell injection, the tumor xenograft model was successfully developed in all injected animals with all tumor volume reached ≥30 mm^3^. Then treatment groups were administrated with different doses of SCS. At week 7 before the sacrifice, the average tumor weight in the model group was 2.311 ± 1.235 g, while the SCS treatment group with low dose and high dose was 2.043 ± 1.001 g and 1.550 ± 0.26 g, respectively (Figure 4F,G). The results of H&E staining showed (Figure 4E) that compared with the model group, the high dose group did not show pronounced tumor necrosis, cell infiltration, and other malignant tumor phenomena.

**Figure 5:**
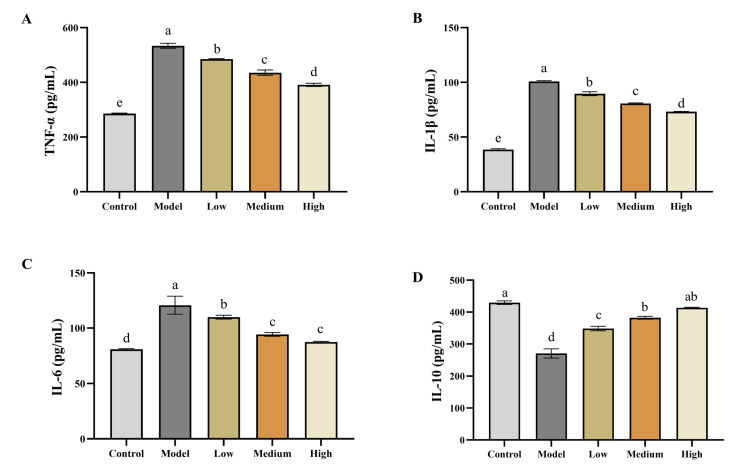

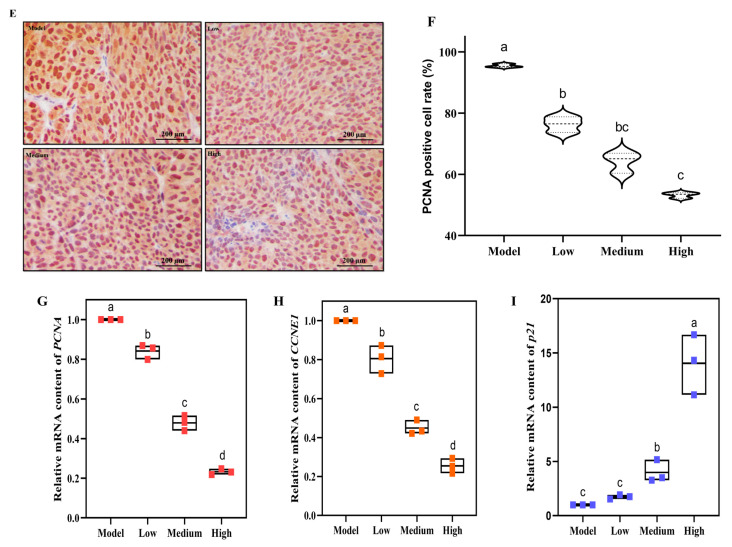
Effect of SCS on regulating cell proliferation pathway in HT-29 xenograft tumor mice model. Total RNA was extracted from xenograft tumor and the gene expression of cell proliferation markers was detected by qPCR. (**A**) Effect of SCS on Tumor Necrosis factor (TNF-α); (**B**) Effect of SCS on Interleukin 1β (IL-1β); (**C**) Effect of SCS on Interleukin 6 (IL-6); (**D**) Effect of SCS on Interleukin 10 (IL-10); (**E**) Xenograft tumor tissues were collected after sacrifice and sectioned for proliferating cell nuclear antigen (PCNA) staining; (**F**) Representation images of PCNA staining; (**G**) The relative mRNA content of PCNA; (**H**) The relative mRNA content of CCNE1; (**I**) The relative mRNA content of P21. All results are expressed as mean ± SD (*n* = 10). Different superscript letters for each column indicated significant differences (*p* < 0.05) as compared to every other group.

Adverse outcomes of cancer are primarily characterized by elevated release of inflammatory cytokines [35]. Therefore, the levels of key pro-inflammatory cytokines (i.e., TNF-a, IL-1B, and IL-6) and anti-inflammatory cytokine (i.e., IL-10) in tumor-bearing mice sera were determined. The results show that after injecting HT-29 cells, the levels of pro-inflammatory cytokines increased in mice sera. After SCS treatment, the levels of those pro-inflammatory cytokines decreased (Figure 5A–C). Treatment with the highest concentration of SCS restored the level of all pro-inflammatory cytokines to close to normal level. IL-10 can activate MAPK/NF-κB cascade in macrophages and plays a role in inducing chromatin remodeling targeting IL-10 promoter. Yu (2019) found that the expression of IL-10 in colon tissue of mice treated with extracellular polysaccharide of *Bifidobacterium* was increased. This is consistent with our results that increase in IL-10 was observed within mice stimulated with SCS treatment [35]. TNF-α, IL-1β and IL-6, as proinflammatory factors, can trigger and inhibit inflammation, thus stimulating adaptive immune response and immune function, also affecting the severity of cancer [33]. In our results, we found that SCS can induce the down-regulation of TNF-α, IL-1β and IL-6 expression, indicating that SCS can inhibit inflammatory response, consistent with RNA-seq predictions.

### 2.9. Mechanisms of SCS Inhibited Colorectal Cancer

#### 2.9.1. SCS Inhibits the Growth of HT-29 by Regulating the Expression of Genes Involved in Tumor Proliferation In Vivo

Proliferating cell nuclear antigen (PCNA) is involved in DNA replication and synthesis, which is an important indicator of cell proliferation [36]. In order to further study the anti-tumor mechanisms of SCS in vivo, we measured the proportion of the tumor cells isolated from the mice that were PCNA positive. Similar to the results *in vitro*, SCS could slow down the proliferation of colorectal cancer in mice. Compared with the model group (95.47 ± 0.17%), PCNA staining showed that the percentage of PCNA positive cells decreased to 76.33 ± 2.60% and 52.32 ± 1.03% in low dose and high dose SCS group, respectively (Figure 5E,F). Consistently, the expression of PCNA in the high dose group was reduced by 5-fold compared to the model group (Figure 5G).

Previous studies also show that *P21* plays a major role in inhibiting cancer progression [20]. For example, Shuang (2015) found that removal of *P21* knockout can directly accelerate the deterioration of liver cancer [37]. *P21* is an inhibitor of cyclin-dependent kinase and multiple studies demonstrated a tumor suppressive role of *P21* in tumorigenesis [38,39]. The main mechanism of *P21* to prevent cell cycle progression is to inhibit the expression of *CCNE1* and *PCNA*, thereby reducing the expression of tumor growth factor and blocking the progress of cell cycle [40,41,42]. In addition to PCNA, we also focused on other two proteins, CCNE1 and P21, that are related to cell proliferation [36]. As shown in Figure 5H,I, SCS treatment induced a down-regulation of *CCNE1* (Figure 5H), showing about a 5-fold reduction after high dose SCS treatment, while SCS treatment resulted in an up-regulation of *P21*. Compared with the model group, high dose group showed about 14-fold increase in *P21* expression (Figure 5I). These results are consistent with the expression pattern observed in our RNA-seq dataset, highlighting the effect of SCS on cell cycle arrest. Collectively, our results show that SCS treatment resulted in cell cycle arrest by inhibiting *PCNA* and *CCNE1* while activating *P21*.

#### 2.9.2. SCS Inhibits the Growth of HT-29 by Promoting Apoptosis of Tumor Cells In Vivo

In addition to the suppression of cell proliferation, the induction of cell apoptosis is another therapeutic approach for cancer treatment. In general, cell apoptosis is regulated by three major pathways: the intrinsic (or mitochondrial), the extrinsic (or death receptor), and the intrinsic endoplasmic reticulum pathways [26]. The process of apoptosis can be divided into four stages: receiving apoptotic signal, interaction between apoptotic regulatory molecules, activation of proteolytic enzyme (Caspase), and entering continuous reaction process. Genes such as *Bcl-2* family genes and *Caspase-8* promote apoptosis. Apoptosis can be induced by activating *Caspase-2*, *3*, *6* [18,29]. In addition, *P53* and *Cytochrome C* also participate in apoptosis by changing membrane permeability [34]. As a cycle regulatory gene, *P21* gene can directly activate apoptosis.

**Figure 6:**
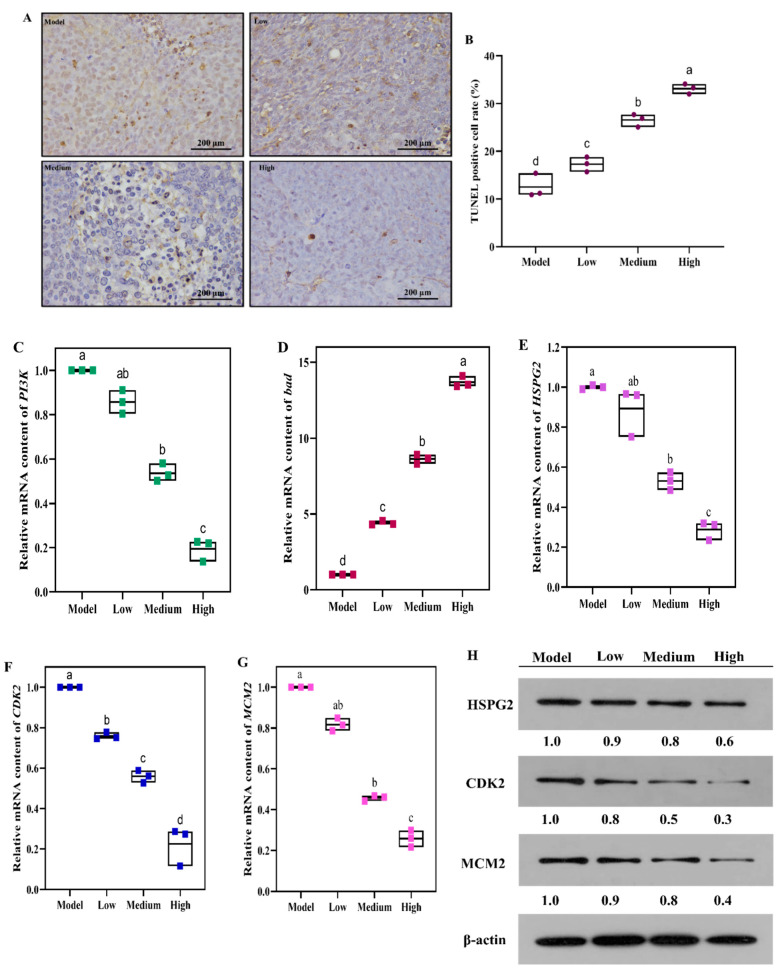
Effect of SCS on cell apoptosis in HT-29 xenograft tumor mice model. (**A**) Representation images of TUNEL staining. Xenograft tumor tissues were collected after sacrifice and sectioned for TdT-mediated dUTP nick-end labeling (TUNEL) staining; (**B**) Percentage of TUNEL-positive cells; (**C**) The relative mRNA content of *PI3K*; (**D**) The relative mRNA content of *bad*; (**E**) the relative mRNA content of *HSPG2*; (**F**) The relative mRNA content of *CDK2*; (**G**) The relative mRNA content of *MCM2*; (**H**) Western blot analyses of HSPG2, CDK2 and MCM2 proteins. Total RNA was extracted from xenograft tumor and the gene expression of cell proliferation markers was detected by RT-PCR, all results are expressed as mean ± SD (*n* = 10). Different superscript letters for each column indicated significant differences (*p* < 0.05) as compared to every other group. Western blot analysis using the indicated antibodies with β-actin as a loading control. The numeric ratios of relative expression were determined by densitometric analyses of the blots using the Image J software.

We found that SCS could induce cell apoptosis in vitro. Transcriptome and 2-DE proteome also showed that apoptosis genes/proteins were differentially expressed in SCS-treated cells. We sought to investigate whether SCS can trigger apoptosis in vivo as well. To address this question, TUNEL staining of tumor tissue was analyzed. The percentage of apoptotic cells in the HT-29 tumors significantly increased to 33.13 ± 0.97% in the high dose group compared to 12.50 ± 2.90% in the model group (Figure 6A,B). In our transcriptome dataset, a series of genes involved in apoptosis were found to be differentially regulated after SCS treatment. PI3K is an intracellular phosphatidylinositol kinase, which participates in the regulation of PI3K/Akt signaling pathway, facilitating cell proliferation, and promotes cell apoptosis [42]. *Bad* is known as a pro-apoptotic protein, which can trigger and promote cell apoptosis but is inactivated in cancer cells [22]. The key genes *PI3K* and *Bad* were selected for RT-qPCR verification. In Figure 6C, it was found that SCS could inhibit the expression of *PI3K*. Furthermore, compared with the model group, *Bad* expression increased about 14-fold in the high dose group (Figure 6D). These results indicate that SCS can activate apoptosis pathway in colorectal cancer by inhibiting *PI3K* while inducing *Bad* expression.

#### 2.9.3. SCS Treatment Results in Cell Cycle Arrest In Vivo

Studies indicated that the multiple mini-chromosome maintenance protein complex, including minichromosome maintenance complex component (MCM2-7), is the core component of replication machinery [43]. It acts as DNA replication helicase to release DNA double strand at the early stage of the cell cycle to initiate DNA replication [43,44]. Previous reports indicated that down-regulation of MCM proteins could induce DNA damage and genome instability in cancer cells, and their dysregulations are also associated with apoptosis. In addition to inhibiting MCM protein complex, there were cyclin-dependent kinase (CDK) inhibitors, which result in cell cycle arrest in G1 phase, and then followed by cell apoptosis [43].

As shown in our results, the effects of SCS can decrease the viability of cancer cells, promote apoptosis, and block cell cycle at G0/G1 phase in vitro and in vivo. We attempted to explore whether SCS treatment in mice changes the expression of several key genes/proteins that regulate colon cancer progression by RT-qPCR and Western blot (Figure 6E–H). Three key genes and their corresponding proteins (HSPG2, CDK2, MCM2) were down-regulated in tumor tissues after SCS treatment. This result is consistent with in vitro RNA-seq and 2-DE analysis. The previous results indicating detection of cell apoptosis after SCS treatment, we speculate that continuous G1 phase block by SCS leads to further cell signal transduction that triggers cell apoptosis.

Surprisingly, through transcriptome analysis, *HSPG2* involved in extracellular matrix (ECM)-receptor interaction was down-regulated after SCS treatment. We observed the same pattern of expression change of HSPG2 in vivo. The results of RT-qPCR showed that the expression level of *HSPG2* was reduced about 3.5-fold after SCS treatment. Western blot showed that SCS treatment reduced HSPG2 production by about 50%. Tumor cells can activate or secrete proteolytic enzymes to degrade the extracellular matrix through the adhesion of their surface receptors with various components of ECM, thus forming a local dissolution zone, which constitutes the tumor cell metastasis operation channel [45,46,47]. The occurrence, development, invasion, and metastasis of malignant tumors are often accompanied by changes in the expression of ECM and its cell surface receptors. *HSPG2* (Heparan Sulfate Proteoglycan 2) is a protein related to integrin pathway and proteoglycans in cancer [48,49,50]. *HSPG2* mainly exists in cancer cells and regulates their proliferation, invasion, and metastasis [51,52]. Our results show that the expression of *HSPG2* decreased after SCS treatment. In addition, studies have shown that HspG2 interacts with laminin to promote the activity of growth factors such as FGF2 [53], thus stimulating the growth and regeneration of endothelial cells. Therefore, we speculate that SCS may inhibit the proliferation of cancer cells by inhibiting the receptor protein (*HSPG2*) on the cell membrane and activating the gene/protein expression in PI3K-Akt signaling pathways.

## 3. Materials and Methods

### 3.1. Material and Reagents

CS in hybrid sturgeon (*Acipenser schrenckii* × *Huso dauricus*) was prepared and preserved in the laboratory. Dulbecco’s modified Eagle medium (DMEM) and phosphate-buffered saline (PBS) were purchased from Gibco (Grand Island, NY, USA). Trypsin-EDTA (0.25 M), paraffin, hematoxylin, and hematoxylin eosin (H&E) staining kit were purchased from Solarbio Technology Co. (Beijing, China). Cell Counting Kit -8 (CCK-8), Hoechst 33258, annexin V-FITC apoptosis detection kit, cell cycle analysis kit, ATP analysis kit and Caspase-3 analysisi kit were purchased from Beyotime Biotechnology (Shanghai, China). Trizol reagent was purchased from Invitrogen (Carlsbad, CA, USA). Reverse transcription kit was purchased from Takara Biotechnology (Dalian, China). Proliferating cell nuclear antigen (PCNA) monoclonal antibody was purchased from Abcam (Cambridge County, UK). TdT-mediated dUTP nick-end labeling (TUNEL) kit was purchased from Beyotime Biotechnology (Shanghai, China). SYBR Fast qPCR kit was purchased from Kapa Biosystems (Indianapolis, IN, USA). Antibodies were purchased from Boster Biological Technology (Wuhan, China).

### 3.2. Cell Culture and Viability Analysis

#### 3.2.1. Cell Viability Assay

Human colorectal cancer cell line HT-29 (ATCC HTB-38), Caco-2 (ATCC HTB-37), HCT-116 (ATCC CRL-247), SW480 (ATCC CCL-228) and LS-174-T (ATCC CL-188) were purchased from the Cell Bank of Chinese Academy of Sciences (Shanghai, China) and cultured in DMEM medium (Gibco, New York, NY, USA) supplemented with 10% of fetal bovine serum (FBS, Every Green, Hangzhou, China) and 1% of penicillin-streptomycin (100 U/mL, Gibco, New York, USA). Human normal colorectal cell line NCM460 (INCELL CVCL_0460) was cultured in DMEM supplemented with FBS 20%. All mammalian cells were incubated at 37 °C in a humidified 5% of CO2 incubator (MCO-15AC, SANYO, Osaka, Japan).

All cells were seeded at a density of 5 × 10^4^ cells/mL into a 96-well plate in DMEM medium for overnight. Then, cells were treated with different concentrations (50, 100, 200, 400, 800, and 1000 μg/mL) of SCS. After 24 h of SCS treatment, 10 μL of CCK8 (Cell Counting Kit -8) was added and cells were incubated for another 4 h. The cell culture medium was mixed on an orbital shaker for 1 min and cell viability was measured using a microplate reader (BioTek, Beijing, China) at 450 nm. The inhibition rate (%) was calculated using the following formula:

Inhibition rate (%) = (control well absorbance − absorbance of the experimental well)/(control well absorbance − blank well absorbance) × 100(1)

#### 3.2.2. Cell Cycle Analysis

Cell cycle analysis was conducted using the Beyotime Biotechnology Cell Cycle Kit and FACScan flow cytometry system (BD Company, New York, NJ, USA). HT-29 and NCM460 cells (1 × 10^5^ cells/well) in 6-well plates were treated with different concentrations (0, 100, 200, and 400 μg/mL) of SCS for 24 h. Proportions of cells in G2/M, S, and G0/G1 phase were quantified. Manufacturer protocols were followed for staining and analysis.

#### 3.2.3. Cell Apoptosis Analysis

HT-29 and NCM460 cells were cultured in 6-well plates at the density of 1 × 10^5^ cells/well and incubated with different concentrations (0, 100, 200, and 400 μg/mL) of SCS. After 24 h, the cells were harvested and re-suspended in195 μL annexin V–FITC binding buffer with addition of 5 μL annexin V–FITC and 10 μL PI solution. The mixture was incubated at room temperature for 20 min in the absence of light. The cells were processed by FACScan flow cytometry system (BD Company, New York, NJ, USA) and analyzed by FCS Express software (De Novo Software, California, CA, USA).

To evaluate membrane integrity and permeability, confocal laser scanning microscopy (CLSM) combined with Hoechst 33258 was used. The cultured cells were treated with different concentrations (0, 100, 200, and 400 μg/mL) of SCS at 37 °C for 24 h. The Hoechst 33258 were incubated at 37 °C for 30 min in the dark. After being washed three times with PBS, images of cell samples were captured using confocal laser scanning microscopy.

#### 3.2.4. Analysis of ATP Level and Caspase-3 Activity

HT-29 and NCM460 cells were cultured in 6-well plates at the density of 1 × 10^5^ cells/well and incubated with different concentrations (0, 100, 200, 400 μg/mL) of SCS. After 24 h, ATP and Caspase-3 were detected by kit and by manufacturer protocols.

### 3.3. RNA-Sequencing (RNA-Seq) and Data Analysis

Total RNA was extracted from the cells with TRIzol reagent according to the manufacturer’s protocol. RNA libraries were prepared using standard Illumina protocols (Illumina). The whole mRNA profile was generated using Illumina HiSeq 2000 platform, and 125-bp paired-end reads were generated. Rraw reads (fastq format) were extracted from Illumina BCL using the Illumina CASAVA program. Clean reads were obtained by removing reads containing adapter and ploy-N and low-quality reads. Cuffdiff was utilized for differential expression analysis [54]. Genes with an adjusted *p*-value < 0.05 detected were assigned as differentially expressed. GO and KEGG annotation were performed for the differential genes using the clusterProfiler package [55].

RNA-seq data were deposited in GenBank under the accession number (GSE179862).

### 3.4. Two-Dimensional Difference Gel Electrophoresis (2D-DIGE)

After being treated with different concentrations of SCS for 24 h, total protein was extracted using a commercial kit (BestBio Company, Shanghai, China). All protein samples were adjusted to the same concentration and subjected to two-dimensional (2-D) gel electrophoresis as described in the literature [56], For electrophoresis, 400 mg of total protein was loaded onto immobilized pH-gradient strips (17 cm, non-linear pH 4–7; Bio-Rad). Isoelectric focusing was performed for 14 h at 50 V, 3.5 h at 150 V, 1 h at 300 V, 1 h at 600 V, 1 h at 1200 V, 4.5 h at 10,000 V, 60,000 Vh at 10,000 V and then maintained at 500 V. Chromatography in the second dimension was performed on 12% SDS-polyacrylamide gel electrophoresis gels. The gels were run for 30 min at 32 mA/2 gel, 6–6.5 h at 64 mA/2 gel, and the electrophoresis was stopped when the bromophenol blue indicator was close to the bottom of the glass plate. After electrophoresis, gels were stained with Colloidal Coomassie Brilliant Blue G-250, and imaged by GS-710 Calibrated Imaging Densitometer (Bio-Rad) scanner. The images were analyzed by PDQuest 8.0 software (Bio-Rad). Protein spots with 1.5-fold change in abundance (*p* < 0.05) were subjected to further identification.

The selected protein spots were de-stained by acetonitrile and then treated with reduction, alkylation, and ultimately digestion using sequencing-grade modified trypsin (034K3707, Sigma-Aldrich, St. Louis, MI, USA). The time-of-flight mass spectrometer (5800 MALDI-TOF/TOF, ab SCIEX) was used for protein identification. The original files of MS were retrieved from the corresponding database by Mascot 2.2 software, and the results of protein identification were obtained. The raw data was searched against National Center of Biotechnology Information (NCBI) protein databases. Predictions of protein function were made based on the Kyoto Encyclopedia of Genes and Genomes (KEGG), Universal Protein (Uniprot) and NCBI.

### 3.5. Animals Experiment and SCS Treatments

Eight-week-old male BALB/c nude mice (Charles River, Beijing, China) were housed in groups of 5 per cage with a 12-h light/12-h dark cycle at 22 ± 2 °C and 65 ± 5% humidity. Food and water were available ad libitum throughout the study. The mice in this study followed the regulation of Assessment and Accreditation of Laboratory Animal Care (AAALAC). All of the procedures were approved by the animal Ethics Committee of Laboratory Animal Science Ethics Committee of Peking University (SYXK (Jing) 2015-0046).

The experimental CRC model was established via HT-29 xenograft. Briefly, HT-29 cells were harvested from cell culture flask and re-suspended at the concentration of 1 × 10^7^ cells/mL. For each of the mice, 100 μL of cell suspension was injected subcutaneously into the right axillary region. The control group received saline instead of tumor cells. When the subcutaneous xenograft tumors volumes reached 30 mm^3^ in diameter, tumor volumes were calculated according to the following formula: Tumor volume (mm^3^) = (length × width^2^)/2. The mice were randomly divided into 5 groups with the following treatments for 4 weeks: (1) Untreated group (Control): tumor-free mice with intragastric administration of saline; (2) Model group (Model): tumor-bearing mice with intragastric administration of saline; (3) High group (400 μg/g): tumor-bearing mice with intragastric administration of 400 μg/g body weight/day of SCS; (4) Medium group (200 μg/g): tumor-bearing mice with intragastric administration of 200 μg/g body weight/day of SCS; (5) Low group (100 μg/g): tumor-bearing mice with intragastric administration of 100 μg/g body weight/day of SCS. The body weight, food intake, and tumor size were measured every week, and the mice’s survival was monitored daily throughout the entire experimental period. In the end of the experiment, all animals were sacrificed and tumors were collected for further analysis. All tumors were bisected, part of them was fixed in 10% formalin and paraffin-embedded for HE staining, and the rest was snap-frozen and stored in liquid nitrogen for RNA and protein extraction. Levels of TNF-α, IL-1β, IL-10, and IL-6 in the sera were detected using individual ELISA kit (Cusabio, Wuhan, China).

### 3.6. Immunohistochemical (IHC) and TUNEL Staining Analysis

Immunohistochemical (IHC) and TUNEL staining were carried out according to the method of Wu (2020) [13]. Sections of 5 μm in thickness were cut from the formalin-fixed tumor samples. Slides were washed three times with PBS and incubated in 5% normal goat serum to block non-specific background staining. Subsequently, the sections were incubated with Rabbit anti PCNA antibody at 4 °C overnight. Then, the sections were washed three times with PBS and incubated with Goat anti rabbit IgG room temperature for 30 min, followed by nuclear re-staining with hematoxylin. Then IHC was observed under microscope.

For TUNEL staining, paraffin sections were dewaxed twice with xylene for 10 min, hydrated with ethanol gradient (100% ethanol for 5 min, 90% ethanol for 2 min, 70% ethanol for 2 min, and ddH2O for 2 min), incubated with proteinase K for 10 min at room temperature, and sealed with 3% H_2_O_2_ in PBS for 20 min at room temperature. Then TUNEL staining was performed according to the manufacturer’s protocol. Briefly, the samples were compared with 50 μL biotin labeling solution (45 μL biotin dUTP and 5 μL TdT) at 37 °C for 60 min with protection against light. The sample was then mixed with 50 μL of 50% streptavidin HRP solution at room temperature for 30 min, followed by incubation in DAB solution at room temperature for 20 min. After being washed with PBS, the slices were re-stained with hematoxylin.

Sections of HIC and TUNEL were examined using AxioVer A1 (Carl Zeiss microscope, New York, NY, USA) at 100× magnification. Image Pro Plus v.6.0 software (Media control Netics, New York, NY, USA) was used to analyze the images.

### 3.7. Quantitative RT-PCR

Total RNA was extracted using TRIzol reagent. cDNA was synthesized using the PrimeScript 1st strand cDNA synthesis kit (Takara, Japan). Quantitative real-time PCR was performed using SYBR Fast qPCR kit (Kapa Biosystems, Wilmington, MA, USA) in 7500 Fast Real-time PCR system (Applied Biosystems, Waltham, CA, USA). The 16S rRNA gene was used as an internal reference gene. The relative quantification of specific genes was analyzed by 2^−ΔΔCt^ method. All primers used in qRT-PCR were listed in Table 2.

**Table 2:** Sequences of primers used in RT-PCR.

Genes	Primer Sequences (5′→3′)
*β* *-action*	F: CGACCACTTTGTCAAGCTCA
	R: AGGGGTCTACATGGCAACTG
*PCNA*	F: TCTGAGGGCTTCGACACCTA
	R: TCATTGCCGGCGCATTTTAG
*p21*	F: AAAGCGCGAACAACTTGACC
	R: GCTGTGCCCTAGAGTGTGTT
*CCNE1*	F: CGGCGAGGGACCAGTGTG
	R: CGGGGAGCCTCTGGATGGT
*PI3K*	F: AGTAGGCAACCGTGAAGAAAAG
	R: GAGGTGAATTGAGGTCC CTAAGA
*bad*	F: CCTTTAAGAAGGGACTTCCTCGCC
	R: ACTTCCGATGGGACCAAGCCTTCC
*HSPG2*	F: GACATCGCCATGGATACCAC
	R: CAGGACAAGCCAGAATAGCC
*CDK2*	F: GCCCTCAAGAGTGTGAGAGTC
	R: CACGAACTGTGCTGATGGGA
*MCM2*	F: ATGATCGAGAGCATCGAGAACC
	R: GCCAAGTCCTCATAGTTCACCA

### 3.8. Western Blot Analysis

Tumor tissues were washed with PBS and treated with cell lysis buffer (Beyotime Biotech, Haimen, Jiangsu, China) containing 1 mM phenylmethylsulfonyl fluoride (PMSF) (Sigma-Aldrich, St. Louis, MO, USA). Protein concentration was determined using bicinchonininc acid (BCA) method. A total of 20 μg of protein from each sample were subjected to 10% SDS-polyacrylamide gel, followed by electrotransferring to PVDF membranes (Millipore, Billerica, MA, USA). These membranes were then washed with Tris-buffered saline supplemented with 0.05% (*v*/*v*) Tween 20 (TBST) and blocked by 5% (*w*/*v*) skimmed milk powder diluted in TBST. The samples were incubated with primary antibodies overnight at 4 °C. After incubation, the membranes were washed five times with TBST and then incubated with secondary antibodies coupled to horseradish peroxidase for 1 h at room temperature. Immunolabeled complexes were detected by enhanced chemiluminescence (ECL) reagents (Millipore, Billerica, MA, USA). Images were obtained by Amersham Imager 600 imaging system (GE Healthcare Life Sciences, Pittsburgh, PA, USA).

### 3.9. Statistical Analysis

All experiments were performed in three biological replicates. The data were analyzed by ANOVA in SPSS 22.0 and PRISM 6 statistical software (GraphPad Software, San Diego, CA). All results are presented as mean ± standard deviation (SD) and *p*-value < 0.05 was considered statistically significant.

## 4. Conclusions

**Figure 7:**
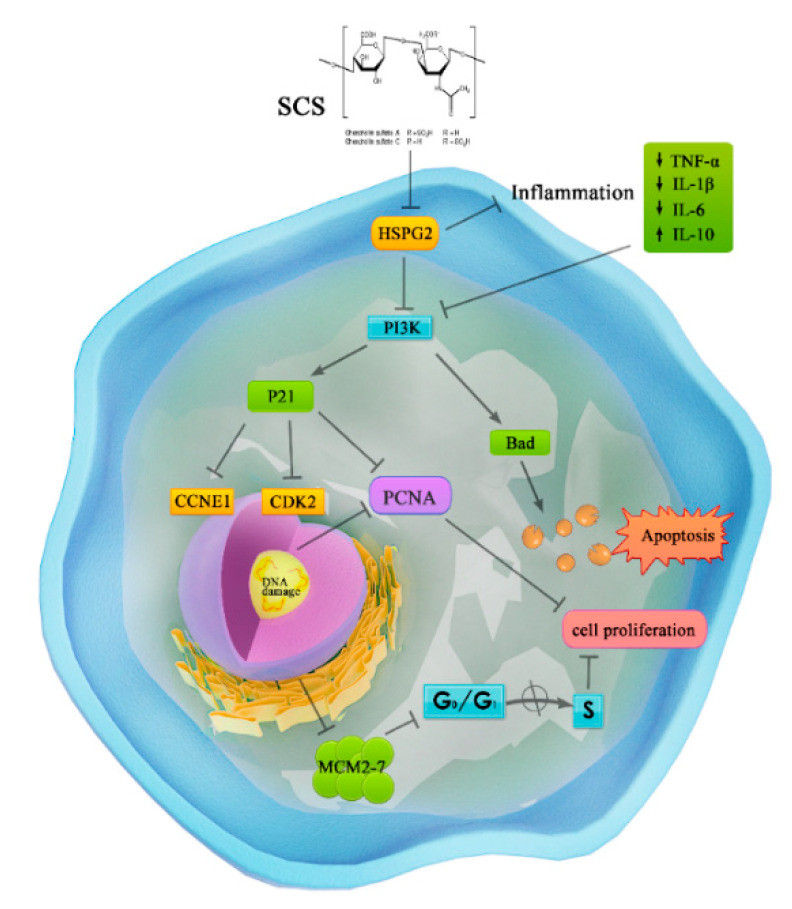
The potential mechanism of SCS-induced inhibition of colorectal cancer progression.

In this study, in vivo and in vitro, SCS can effectively inhibit the proliferation of colorectal cancer cell line HT-29. SCS treatment showed little damage to normal intestinal epithelial cell line. Using transcriptomics and proteomics, we identified genes/proteins associated with cell cycle and apoptosis were impacted by SCS treatment in vitro. Consistently, our in vivo results show that SCS-inhibited tumor growth mainly inhibits the proliferation of cancer cells by inhibiting the receptor protein (HSPG2) on the cell membrane and activating the gene/protein expression in PI3K-Akt signaling pathways, promoting the expression of apoptosis-related genes (Bad) and blocking cell cycle (P21, CDK2, CCNE1 and MCM2) (Figure 7). The findings of this study can provide great insights into the development of therapeutic drugs for colorectal cancer from food-derived sources.

## Figures and Tables

**Table 1:** Identification of differentially expressed proteins of SCS treatment in HT-29/NCM460.

Spot	Gene Name	Score	*M*_W_	PI	F.C	Accession No.
**HT-29 (0–100)**						
927	ATP synthase	168	15,820.2	6.6	0.40	gi|119609635
689	cyclin-dependent kinase 2 (CDK2)	608	33,000.0	5.08	0.17	gi|1017
1224	proliferating cell nuclear antigen (PCNA)	516	36,026.6	4.63	0.22	gi|5111
677	BCL2 associated agonist of cell death (Bad)	516	22,101.3	6.13	6.00	gi|527
1452	BCL2 binding component 3 (BBC3)	425	22,992.3	5.12	4.80	gi|27113
1085	heterogeneous nuclear ribonucleoprotein C	415	33,706.6	4.95	3.00	gi|119586799
1448	DNA-damage-inducible transcript 4 (DDIT4)	199	26,200	7.6	3.33	gi|54541
1374	eukaryotic translation elongation factor 1 gamma	631	50,429.3	6.25	2.50	gi|119594431
1261	minichromosome maintenance complex component 2 (MCM2)	309	124,759.6	5.82	0.33	gi|4171
709	Human Hsp27	290	10,383.2	5.75	2.80	gi|609412402
**NCM460 (0–100)**						
1420	6-phosphogluconolactonase	262	27,814.7	5.7	0.46	gi|6912586
767	heterogeneous nuclear ribonucleoprotein	535	48,760.2	5.38	5.00	gi|530391069

## Data Availability

https://www.ncbi.nlm.nih.gov/geo/query/acc.cgi?acc=GSE179862.

## Supplementary Materials

The following are available online at https://www.mdpi.com/article/10.3390/ijms22179395/s1.

## Abbreviations

ANOVA	One-way analysis of variance
CCK-8	Cell Counting Kit-8
CRC	Colorectal cancer
CS	Chondroitin sulfate
DMEM	Dulbecco’s modified eagle medium
FBS	Fetal bovine serum
FITC	Fluorescein isothiocyanate
IHC	Immunohistochemical
H&E	hematoxylin eosin
PBS	Phosphate buffered saline
PCNA	Proliferating Cell Nuclear Antigen
PI	Propidium iodide
qPCR	Quantitative real-time polymerase chain reaction
SCS	Sturgeon chondroitin sulfate
SD	Standarddeviation
TdT	Terminal deoxynucleotidyl transferase
TUNEL	TdT-mediated dUTP nick-end labeling
ECM	extracellular matrix
RNA-Seq	RNA-Sequencing
2D-DIGE	Two-dimensional Difference Gel Electrophoresis
GEO	Gene Expression Omnibus
KEGG	Kyoto Encyclopedia of Genes and Genomes
NCBI	National Center of Biotechnology Information
