# Supplementary material for: The Regulatory Network of Sturgeon Chondroitin Sulfate on Colorectal Cancer Inhibition by Transcriptomic and Proteomic Analysis
Source: Int J Mol Sci. 2021 Aug 30;22(17):9395. doi: 10.3390/ijms22179395 (PMC8430666; doi:10.3390/ijms22179395)
Supplement: Supplementary file 1 [file ijms-22-09395-s001.zip › ijms-1333721-Supplementary data.pdf]

Supplementary data

The regulatory network of sturgeon chondroitin sulfate on col-orectal cancer inhibition by transcriptomic and proteomic analysis

Ruiyun Wu [1], Qian Shen [2], Guangyue Li [3], Pinglan Li [1,*] and Nan Shang [4,*]

[1] Key Laboratory of Precision Nutrition and Food Quality, Key Laboratory of Functional Dairy, Ministry of Education; College of Food Science and Nutritional Engineering; China Agricultural University, Beijing 100083; wry0814@cau.edu.cn (R.W.); lipinglan@cau.edu.cn (P.L.)

[2] Rhodes College, Department of Biology, 2000 North Pkwy, Memphis, TN 38112; shenq@rhodes.edu

[3] Key Laboratory of Saline-alkali Vegetation Ecology Restoration, Ministry of Education, College of Life Science, Northeast Forestry University, Harbin, 150006; guangyuel@nefu.edu.cn

[4] College of Engineering, China Agricultural University, Beijing 100083; nshang@cau.edu.cn

* Correspondence: lipinglan@cau.edu.cn (P.L.); nshang@cau.edu.cn (N.S.);

Tel./Fax: +86-010-6273-8678 (P.L.)

Figure S1.

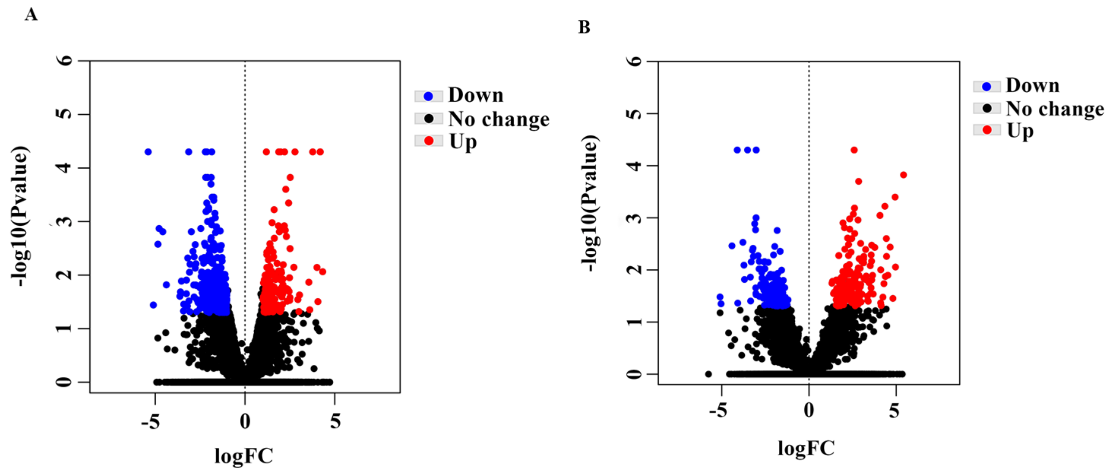

Figure S1. Volcano plots in the two datasets. (A) HT-29 cells; (B) NCM460 cells.

Figure S2.

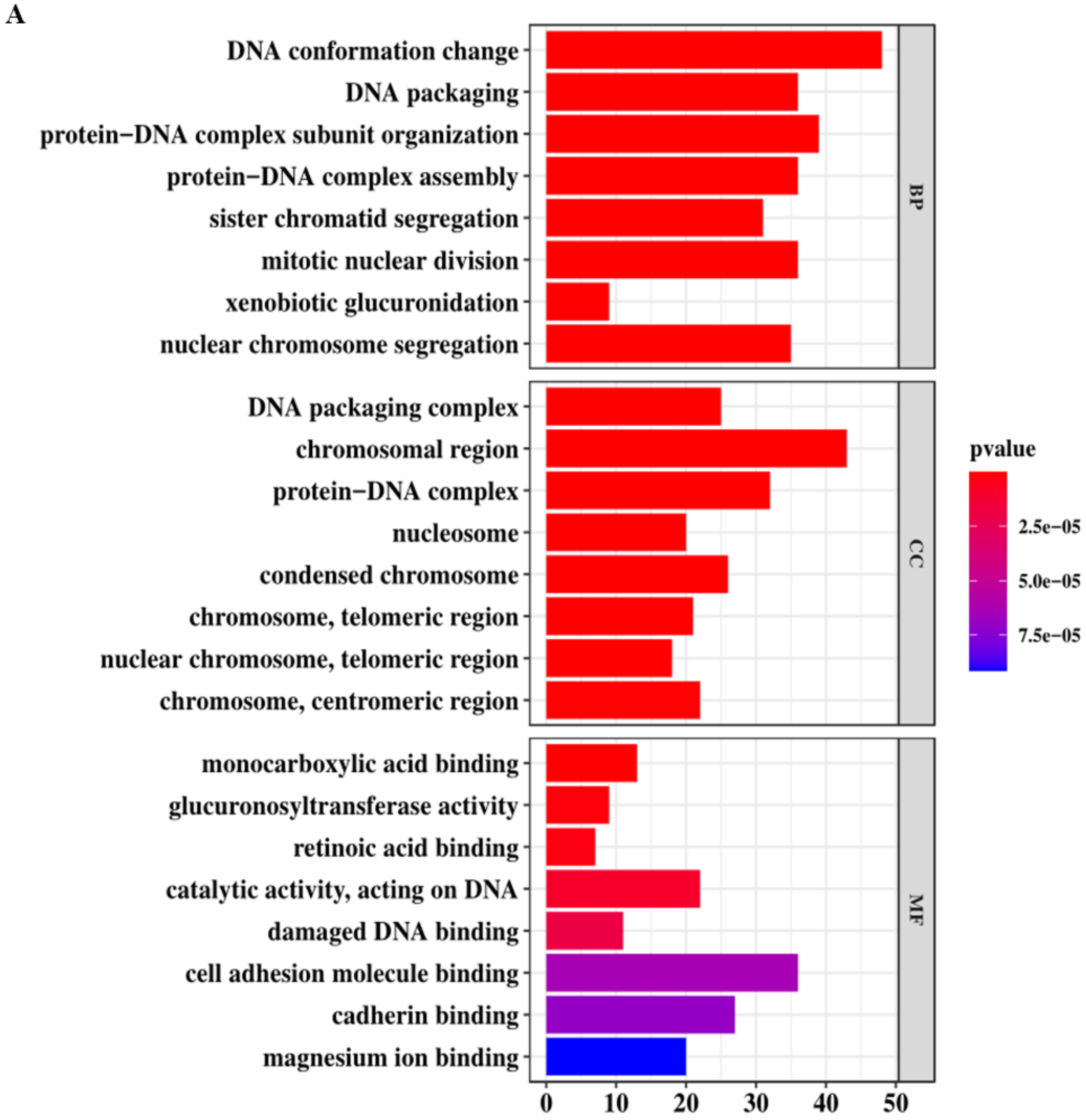
A
DNA conformation change
DNA packaging
protein−DNA complex subunit organization
protein−DNA complex assembly
sister chromatid segregation
mitotic nuclear division
xenobiotic glucuronidation
nuclear chromosome segregation
BP
DNA packaging complex
chromosomal region
protein−DNA complex
nucleosome
condensed chromosome
chromosome, telomeric region
nuclear chromosome, telomeric region
chromosome, centromeric region
CC
monocarboxylic acid binding
glucuronosyltransferase activity
retinoic acid binding
catalytic activity, acting on DNA
damaged DNA binding
cell adhesion molecule binding
cadherin binding
magnesium ion binding
MF
0
10
20
30
40
50
pvalue
2.5e−05
5.0e−05
7.5e−05

B

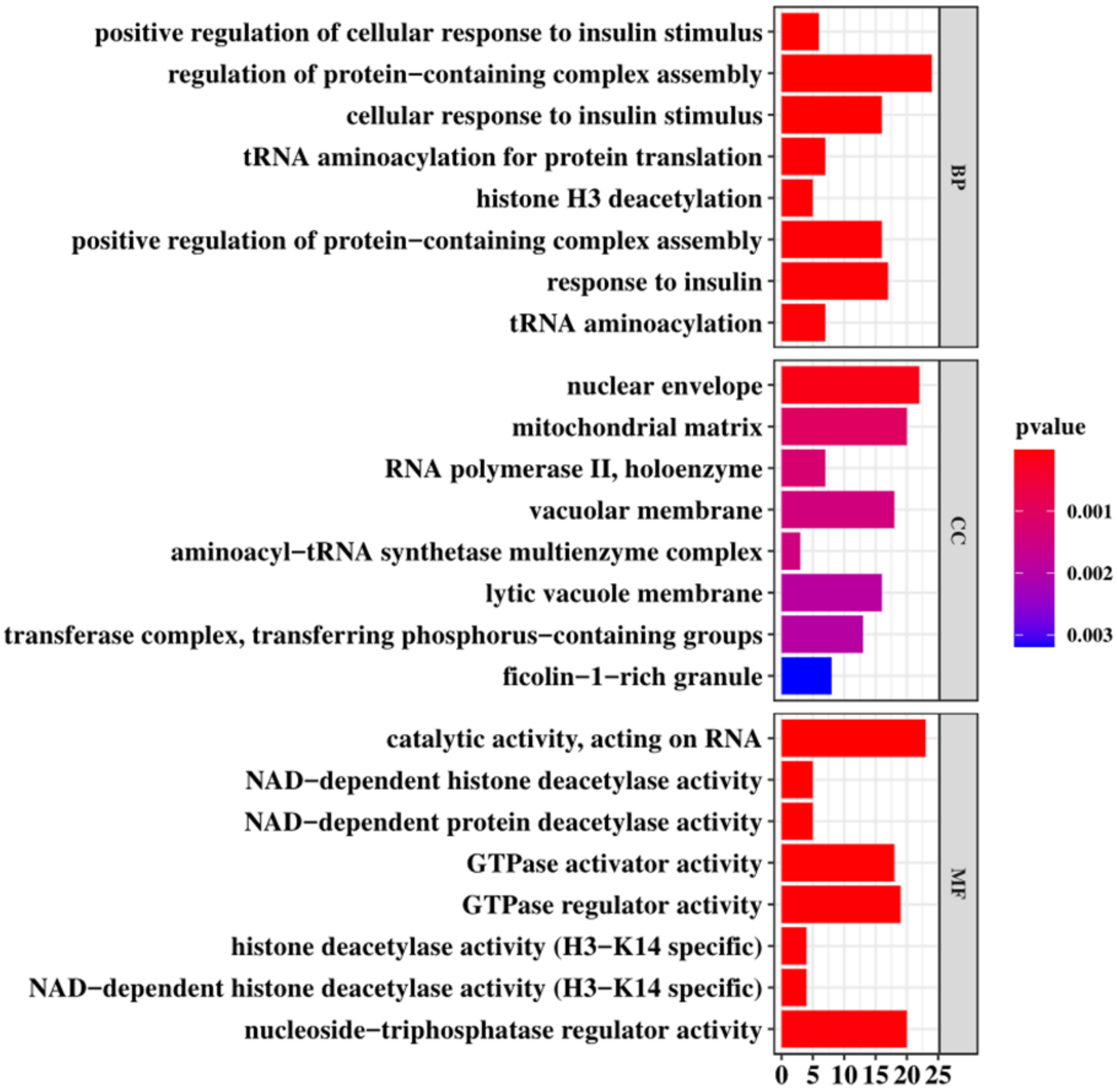

Figure S2. The analyses of the genes with reversely changed expression between the two datasets by the GO enrichment analyses. (A) In HT-29 cells treated with SCS; (B) In NCM460 cells treated with SCS.

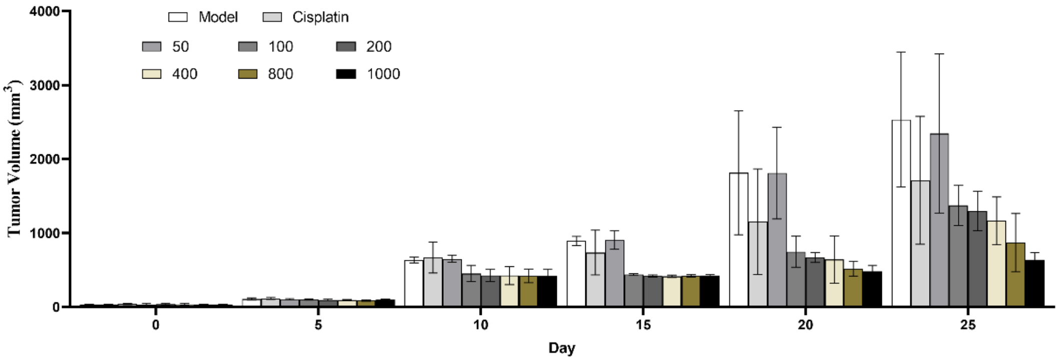

Figure S3. Effect of SCS in HT-29 xenograft tumor mice model. Colon cancer HT-29 cells were injected into BALB/c nude mice to develop the CRC model, while the control group was injected with normal saline, positive control group was given cisplatin by gavage, other mice received an intragastric administration of different doses (50, 100, 200, 400, 800 and 1000 μg/g/day) of SCS (spine) for 25 days.

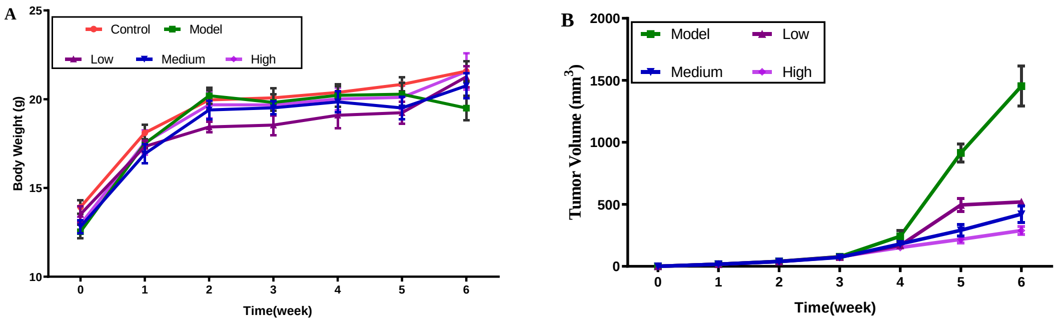

Figure S4. Effect of SCS on body weight in HT-29 xenograft tumor mice model. Colon cancer HT-29 cells were injected into BALB/c nude mice to develop the CRC model, while the control group was injected with normal saline. After 3 weeks, mice received an intragastric administration of different doses (100, 200, and 400 μg/g/day) of SCS (spine) for 4 weeks. (A) body weight, (B) Tumor volume in each time point. All results are expressed as mean ± SD (n = 10).

Table S1. Genes were up- and down-regulated in the transcriptome of SCS treatment in HT-29 (FoldChange > 1.5, $P < 0.05$).

Gene_ID	Gene	Fold change	Fpkm (HT-29-0)	Fpkm (HT-29-100)	Description
ENSG00000197150	ABCB8	0.849971	6.2411	11.2494	ABC transporters
ENSG00000117528	ABCD3	-1.07049	18.9982	9.04614	ABC transporters
ENSG00000076555	ACACB	-4.84956	6.25005	0.21678	Pyruvate metabolism
ENSG00000114331	ACAP2	0.597227	9.66951	14.6281	Endocytosis
ENSG00000151726	ACSL1	0.387771	11.3748	14.8824	PPAR signaling pathway
ENSG00000131069	ACSS2	-0.231053	38.87	33.1177	Pyruvate metabolism
ENSG00000170017	ALCAM	1.23344	3.46587	8.1492	Cell adhesion molecules
ENSG00000137124	ALDH1B1	-0.81218	6.42896	3.66143	Pyruvate metabolism
ENSG00000143153	ATP1B1	-0.807001	134.566	76.9139	cAMP signaling pathway
ENSG00000168646	AXIN2	-1.16822	20.2617	9.01586	Colorectal cancer
ENSG00000105327	BBC3	0.539745	15.3826	22.3619	Apoptosis

ENSG00000089685	BIRC5	-0.84584	23.1477	12.8791	Apoptosis
ENSG00000007516	BAIAP3	1.3484	2.2313	5.68157	Transcriptional misregulation in cancer
ENSG00000125378	BMP4	-0.819866	126.798	71.8305	Cytokine-cytokine receptor interaction
ENSG00000067191	CACNB1	0.324423	1.92517	2.41063	MAPK signaling pathway
ENSG00000129007	CALML4	-0.7125	69.6108	42.4808	cAMP signaling pathway
ENSG00000105974	CAV1	-2.61951	7.09201	1.15403	Endocytosis
ENSG00000134057	CCNB1	-1.68013	49.5962	15.4767	Cell cycle
ENSG00000112576	CCND3	-0.585199	24.5289	16.3499	Cell cycle
ENSG00000105173	CCNE1	-0.97821	13.0678	6.63332	Cell cycle
ENSG00000123374	CDK2	-1.149	19.1165	8.62033	Cell cycle
ENSG00000124762	CDKN1A	1.78979	8.10451	28.0191	Colorectal cancer
ENSG00000109861	CTSC	-0.446879	54.5608	40.0273	Apoptosis
ENSG00000103811	CTSH	-0.507619	96.7967	68.0851	Apoptosis

ENSG00000135047	CTSL	0.728868	2.31248	3.83256	Apoptosis
ENSG00000101160	CTSZ	-0.70697	116.913	71.6218	Apoptosis
ENSG00000134574	DDB2	-1.301	10.7152	4.34871	p53 signaling pathway
ENSG00000168209	DDIT4	1.53455	13.8282	40.06	PI3K-Akt signaling pathway
ENSG00000280962	DUSP16	-0.318543	10.8513	8.70145	MAPK signaling pathway
ENSG00000101412	E2F1	-1.74053	12.1646	3.64036	Cell cycle
ENSG00000100664	EIF5	1.37433	18.6057	48.2347	RNA transport
ENSG00000049283	EPN3	-0.47439	16.4876	11.8673	Endocytosis
ENSG00000181104	F2R	-0.877261	32.0981	17.4742	PI3K-Akt signaling pathway
ENSG00000168496	FEN1	-2.26945	45.3488	9.40576	DNA replication
ENSG00000068078	FGFR3	-1.53518	5.16782	1.78308	PI3K-Akt signaling pathway
ENSG00000160867	FGFR4	-1.41976	6.2622	2.34065	PI3K-Akt signaling pathway
ENSG00000170345	FOS	-1.0351	202.181	98.6609	Apoptosis

ENSG00000001084	GCLC	0.988895	28.9117	57.3801	Ferroptosis
ENSG00000023909	GCLM	0.743281	8.6737	14.5196	Ferroptosis
ENSG00000130513	GDF15	1.50658	129.692	368.502	Cytokine-cytokine receptor interaction
ENSG00000082516	GEMIN5	-0.868009	5.60923	3.07331	RNA transport
ENSG00000127955	GNAI1	-1.39683	11.6652	4.43002	Rap1 signaling pathway
ENSG00000106070	GRB10	1.35392	6.71316	17.1592	mTOR signaling pathway
ENSG00000075218	GTSE1	-0.848843	8.26027	4.58635	p53 signaling pathway
ENSG00000196917	HCAR1	-0.354228	14.8297	11.6011	cAMP signaling pathway
ENSG00000113161	HMGCR	-0.595807	30.1156	19.9267	AMPK signaling pathway
ENSG00000142798	HSPG2	-0.0932491	36.616	34.3241	Focal adhesion
ENSG00000138413	IDH1	1.07213	45.2337	95.1055	Central carbon metabolism in cancer
ENSG00000185885	IFITM1	-0.374148	76.3839	58.9348	B cell receptor signaling pathway
ENSG00000008517	IL32	inf	0	1.15016	Cytokine-cytokine receptor interaction

ENSG00000169047	IRS1	-0.232343	14.7932	12.5927	PI3K-Akt signaling pathway
ENSG00000091136	LAMB1	-0.320342	14.1754	11.3528	Focal adhesion
ENSG00000130164	LDLR	-0.0835284	30.7943	29.062	Endocytosis
ENSG00000105486	LIG1	-2.02756	39.3373	9.64822	DNA replication
ENSG00000113368	LMNB1	-2.20548	65.7346	14.2521	Apoptosis
ENSG00000176619	LMNB2	-0.395009	88.6966	67.4524	Apoptosis
ENSG00000101577	LPIN2	0.918791	14.4732	27.3621	mTOR signaling pathway
ENSG00000164109	MAD2L1	-1.36335	24.2475	9.42451	Cell cycle
ENSG00000140941	MAP1LC3B	0.86672	26.3422	48.0354	Ferroptosis
ENSG00000095015	MAP3K1	-1.0113	13.9454	6.91829	MAPK signaling pathway
ENSG00000107968	MAP3K8	-1.5223	15.7494	5.48284	MAPK signaling pathway
ENSG00000188130	MAPK12	-0.373649	17.7241	13.68	MAPK signaling pathway
ENSG00000073111	MCM2	-1.54024	46.1521	15.8684	Cell cycle

ENSG00000112118	MCM3	-1.83212	30.0111	8.42866	Cell cycle
ENSG00000104738	MCM4	-1.68884	55.6279	17.2545	Cell cycle
ENSG00000100297	MCM5	-1.79599	29.4571	8.48287	Cell cycle
ENSG00000076003	MCM6	-1.93916	15.6355	4.07726	Cell cycle
ENSG00000085276	MECOM	-0.678179	9.34007	5.83711	MAPK signaling pathway
ENSG00000099875	MKNK2	1.52839	37.9716	109.535	MAPK signaling pathway
ENSG00000095002	MSH2	-0.979808	16.243	8.23597	Colorectal cancer
ENSG00000065534	MYLK	-1.16018	6.26297	2.80241	Apelin signaling pathway
ENSG00000155561	NUP205	0.124883	17.4242	18.9997	RNA transport
ENSG00000132182	NUP210	-0.630834	23.6251	15.2572	RNA transport
ENSG00000213024	NUP62	-0.82211	42.2889	23.9192	RNA transport
ENSG00000143799	PARP1	-1.43	61.1417	22.6916	Apoptosis
ENSG00000132646	PCNA	-1.73915	98.694	29.5635	DNA replication

ENSG00000145431	PDGFC	-1.82629	15.0376	4.24044	Rap1 signaling pathway
ENSG00000155629	PIK3AP1	-1.43272	6.52062	2.41544	PI3K-Akt signaling pathway
ENSG00000145675	PIK3R1	-0.182374	10.5984	9.33982	PI3K-Akt signaling pathway
ENSG00000127564	PKMYT1	-2.00423	16.4128	4.0912	Cell cycle
ENSG00000105499	PLA2G4C	1.95771	2.05426	7.97971	Necroptosis
ENSG00000129219	PLD2	-0.609837	5.8466	3.83111	cAMP signaling pathway
ENSG00000166851	PLK1	-0.59503	38.7316	25.6415	Cell cycle
ENSG00000062822	POLD1	-1.22843	32.7651	13.9835	DNA replication
ENSG00000177084	POLE	-1.43174	28.457	10.5486	DNA replication
ENSG00000148229	POLE3	-1.56145	23.1675	7.84937	DNA replication
ENSG00000148737	TCF7L2	-0.673697	29.4119	18.4383	Adherens junction
ENSG00000160293	VAV2	-1.12558	26.4165	12.1072	Focal adhesion

Table S2. Genes were up- and down-regulated in the transcriptome of SCS treatment in NCM460 (FoldChange > 1.5, $P < 0.01$).

Gene_ID	Gene	Fold change	Fpkm (NCM460-0)	Fpkm (NCM460-100)	Description
ENSG00000117528	ABCD3	2.58858	1.53185	9.21413	ABC transporters
ENSG00000114331	ACAP2	5.42009	0.18855	8.07303	Endocytosis
ENSG00000062822	POLD1	-1.81149	40.939	11.6634	DNA replication
ENSG00000166851	PLK1	-1.67534	65.2857	20.4405	Cell cycle
ENSG00000105486	LIG1	-1.77395	24.4869	7.16011	DNA replication

Table S3. Effect of SCS on cell apoptosis in HT-29 and NCM460 cells. The apoptosis ratios (%) were calculated by cell apoptosis assay kit and flow cytometer.

	Annexin V/PI	Control	100 μg/mL	200 μg/mL	400 μg/mL
HT-29	-/-	85.69±0.11	63.43±2.99	50.65±2.33	16.61±0.31
	+/-	7.94±0.09	23.70±1.54	31.08±2.92	64.75±2.98
	+/+	6.33±0.18	12.85±1.88	11.53±1.43	18.29±4.04
	-/+	0.037±0.013	0.017±0.01	0.07±0.04	0.35±0.53
NCM460	-/-	88.32±0.26	81.00±1.79	81.56±6.16	75.91±0.59
	+/-	5.48±2.00	10.52±0.55	13.11±1.08	14.68±0.05
	+/+	6.07±0.10	8.45±0.94	8.36±0.41	9.55±0.69
	-/+	0.12±0.18	0.027±0.006	0.013±0.003	0.023±0.007

Values are expressed as mean ± SD.

Statistical analysis of differences between groups was performed using one-way ANOVA.
